# Antiangiogenic and Proapoptotic Activities of Atorvastatin and Ganoderma lucidum in Tumor Mouse Model via VEGF and Caspase-3 Pathways

**DOI:** 10.31557/APJCP.2021.22.4.1095

**Published:** 2021-04

**Authors:** Iman Hesham El-Khashab

**Affiliations:** *Department of Zoology, Faculty of Women for Arts, Science and Education, Ain Shams University, Cairo, Egypt. *

**Keywords:** Caspase-3, VEGF, apoptosis, Atorvastatin, Ganoderma lucidum

## Abstract

**Background:**

The statin drug Atorvastatin (AT) used for cholesterol reduction and Ganoderma lucidum (Gl) mushroom extract exhibited satisfactory antitumor activities towards various types of cancer.

**Objective:**

The present study was designed to evaluate the apoptotic and antiangiogenic effects of Atorvastatin and/or Ganoderma lucidum against Ehrlich solid tumor inoculated in female mice.

**Materials and Methods:**

Atorvastatin (AT) or/and Ganoderma lucidum (Gl) extract were administered to mice bearing tumor alternatively for 28 days after 10 days of tumor cells inoculation. Mice were divided into 5 equal groups as follows: Control (C): Normal mice, Ehrlich (E): mice injected in thigh with EAC cells, (E+AT): mice bearing solid tumor that received an intraperitoneal dose of Atorvastatin (10 mg/kg). Group (4): (E+Gl): mice bearing solid tumor that received an oral dose of Ganoderma lucidum (28 mg/kg) Group (5): (E+AT+Gl): mice bearing solid tumor that received intraperitoneal dose of Atorvastatin and oral dose of Ganoderma lucidum.

**Results:**

showed that administration of Atorvastatin and/or Ganoderma lucidum to mice bearing tumor, reduced tumor size, increased MDA level and decreased GSH, SOD and CAT levels in tumor tissues. Histopathological study showed high attenuation in tumor cells associated with antiangiogenesis illustrated by extravasation of blood vessels between tumor cells. Immunohistochemical study demonstrated high reduction of the angiogenic marker Vascular endothelial growth factor (VEGF) with remarkable increase of the apoptotic protein markers cytochrome-c and caspase-3.

**Conclusion:**

Atorvastatin and Ganoderma lucidum may have anticancer, apoptotic and antiangiogenic activities by reducing tumor growth in Ehrlich solid tumor. Their antitumor effect is exerted through the antiangiogenesis effect in tumor cells which is confirmed by the decrease of the angiogenic marker (VEGF protein) as well as by inducing significant increase in the apoptotic protein markers cytochrome-c and caspase-3. It is noticeable that the antitumor activity is ameliorated by the combination of the two treatments.

## Introduction

Cancer is one of the most severe diseases where cells undergo abnormal uncontrolled cell growth division leading to the development of a tumor (Stewart et al., 2016). Tumor cells have crucial behaviors which contribute to their survival; loss of regulated cellular differentiation, loss of regulated cell proliferation, abnormal cell division, inhibition of apoptosis and angiogenesis (Hanahan and Weinberg, 2000). Angiogenesis is a process involving the growth of new blood vessels from pre-existing vessels, contributing to the progression and development of various pathological conditions including tumor growth (Griffioen and Molema, 2000). As tumor growth and development depend on the growth of tumor blood vessels through tumor angiogenesis therefore, targeting tumor angiogenesis is essential for controlling tumor growth (Kobayashi and Lin, 2006). The Ehrlich tumor is a spontaneous mammary tumor and a model of solid tumor in mice (Segura et al., 2000). Statins are drugs reducing the activity of cholesterol levels by inhibiting 3-hydroxy-3-methylglutaryl coenzyme A (HMG Coenzyme A) reductase which is the key enzyme in cholesterol synthesis (Hindler et al., 2006). Statins are mainly used in cardiovascular diseases. However, they are widely recognized by their anticancer activities mediated by proapoptotic and antiproliferative properties (Adnan et al., 2017). Statins target HMG Coenzyme A and increase tumor apoptosis by inhibiting tumor growth inducing cell cycle arrest (Stine et al., 2016). Atorvastatin (AT) is a statin drug that lowers cholesterol production thus preventing cardiovascular diseases (Pisanti et al., 2014). It is the most widely approved statin used for cholesterol reduction (Chen et al., 2016). Previous studies indicated that AT may be associated to reduce cancer developing by inhibiting tumor growth in breast, pancreatic, prostate, and liver cancer and became a potent anticancer drug (Brown et al., 2012). Fungi are a vital source of foods and drug precursors, exhibiting a wide range of pharmacological activities such as anticancer, anti-inflammatory and antitumor effects. (Wu et al., 2014). About 10,000 known species of mushrooms, 2000 of them are safe for people health and nearly 300 species possess medical properties (Wasser and Weis, 1999). Ganoderma lucidum is a type of mushroom with various bioactive compounds, among the Japanese it is called Reishi (Cao et al., 2012). Recent clinical and pharmacological investigations established that Ganoderma lucidum is a natural medicine widely used in Asian countries; it has an essential antitumor activity suppressing tumor cells through its anti-angiogenic, anti-proliferative and pro-apoptotic effects (Sohretoglu and Huang, 2018). The aim of this work is to investigate the antitumor and apoptotic activities of Atorvastatin and/or Ganoderma lucidum extract by biochemical, histolological and immunohistochemical (VEGF, cytochrome-c and caspase-3) investigations in Ehrlich solid tumor bearing mice.

## Materials and Methods

*Animals categorization*

In this experimental study, 50 adult female Swiss albino mice weighing (22-25 g) were obtained from the breeding unit of the Egyptian Organization for Biological Products and Vaccines (Cairo).

Animal procedures were performed in accordance with the principals and guidelines for the care and use of experimental animals of the National Institutes of Health (NIH) protocol. 

The animals were acclimatized for one week and received food and water ad libitum with daily fresh supplies. 

*Drugs and chemicals*

Atorvastatin (AV): was obtained from Pfizer Pharmaceutical Company (Egypt). 

Ganoderma lucidum: obtained from USA Solaray^® ^incorporated. 

Ehrlich solid carcinoma tumor model: This is a spontaneous mammary tumor in mice. It may evolve to solid form depending on the route of inoculation (intraperitoneal or subcutaneous respectively) (Silva et al., 2002). In this study, the tumor was maintained in the solid form in Swiss mice). Solid Ehrlich tumor model was used, where Ehrlich Ascites Carcinoma cells obtained from the Pharmacology and Experimental Oncology Unit of the National Cancer Institute, Cairo University, Egypt were implanted subcutaneously into the right thigh of the lower limb of mice. A palpable solid tumor mass of 1 cm in diameter is obtained approximately within 10 days (Osman et al., 1993). Solid tumor was induced by injecting Ehrlich Ascites Carcinoma EAC cells (2X106 cells/animal). Each mouse was injected with 0.2 ml saline suspension of 2.5 × 10^6^ EAC/ml subcutaneously to the right hind limb of the animal (Fahim et al., 1997). 

*Experimental design:*

A total of 50 female albino mice were divided into 5 equal groups, 10 mice each.

Group (1): (C): Normal skeletal muscles of the control group, where mice served as negative control. Group (2): Ehrlich (E): 0.2 ml (2.5x 106 EAC cells) was inoculated subcutaneously in the right thigh of the lower limb of each female mouse to induce Ehrlich Solid Tumor (EST), (Gupta et al., 2004). and is served as positive control. Group (3): (E+AT): Mice bearing solid tumor that received an intraperitoneal dose of Atorvastatin (10 mg/kg) on three alternative days for 28 days after 10 days of EAC cell inoculation according to Paula et al., (2018). Group (4): (E+Gl): Mice bearing solid tumor that received an oral dose of Ganoderma lucidum (28 mg/kg) by stomach tube on three alternative days for 28 days after 10 days of EAC cell inoculation according to Sohretoglua and Huang (2018). Group (5): (E+AT+Gl): Mice bearing solid tumor that received intraperitoneal dose of Atorvastatin (10 mg/kg) and oral dose of Ganoderma lucidum (28 mg/kg) on three alternative days for 28 days after 10 days of EAC cell inoculation.Tumor tissues were collected for biochemical, histopathological and immunohistochemical investigations.

*Monitoring the tumor size*

Tumor volume was measured on the 28th day after tumor cell inoculation using a caliper and calculated by applying the equation described by Nagle et al. (2004). 

Tumor volume (mm^3^) = 4/3(1/2 smaller diameter)^2^ (1/2 larger diameter).

*Biochemical analyses*

Solid tumor was homogenized in phosphate buffer saline (10 %) and used for various biochemical analyses. Malondialdehyde level (MDA) was evaluated as an indication to lipid peroxidation according to the method described by Yoshioka et al., (1979). Reduced glutathione (GSH) was assessed according to Beutler et al., (1963). Superoxide dismutase (SOD) and catalase (CAT) activities were estimated according to Minami and Yoshikawa (1979); Aebi (1984), respectively. 

*Histopathological investigation*

Samples were fixed in10% formalin, embedded in paraffin and stained with hematoxylin and eosin. Later the slides were examined under the light microscope (Bancroft et al., 2012). 

*Immunohistochemical investigations*

Immunohistochemical staining was performed in tumor tissue with 4 lm-thick sections that were deparaffinized and incubated with fresh 0.3% hydrogen peroxide in methanol for 30min at room temperature. Briefly, deparaffinized tissue slides were incubated with the antibodies against VEGF antibody (diluted at 1:200), cytochrome-c antibody (diluted at 1:200) and caspase-3 antibody (diluted at 1:200)

The antibody binding sites was visualized with 3, 3’diaminobenzidine. The sections were counterstained with hematoxylin, then dehydrated using graded alcohols and xylene, and mounted. The immunostaining intensity and cellular localization of VEGF, cytochrome-c and caspase-3 were analyzed by light microscopy (Cuello, 1993). For immunohistochemistry quantification, the positive area percentage (%) of positive brown stained cells was estimated using TinEye Lab software.

*Statistical analysis*

Data were subjected to statistical analysis and tests of significance were performed using the statistical package SPSS (Statistical Program for Social Science) version

20.0 (SPSS, Chicago, IL) by applying a one-way ANOVA test followed by post hoc test for multiple comparisons. All data are expressed as a mean of 6 values Standard Error and the difference between means are considered significant at p<0.05.

## Results

*Tumor volume (mm* ^3^ *)*

**Figure 1:**
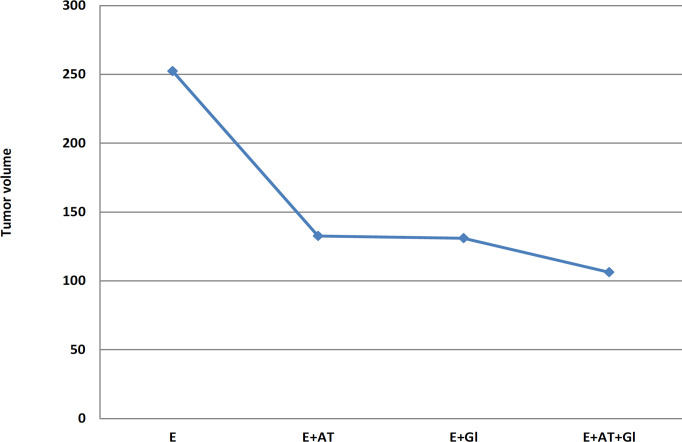
Effect of AT and/or Gl on Tumor Volume

Volume of solid tumor is measured by caliper in mice of the Ehrlich group and the Ehrlich treated with AT and /or Gl on the 28th day. Measurement of tumor volume revealed an extremely significant decrease in its value among animals bearing tumor treated with both AT and Gl than in each treatment alone (Table 1) and (Figure 1).

*Assessment of lipid peroxidation and some antioxidant enzymes in tumor tissue*

**Figure 2:**
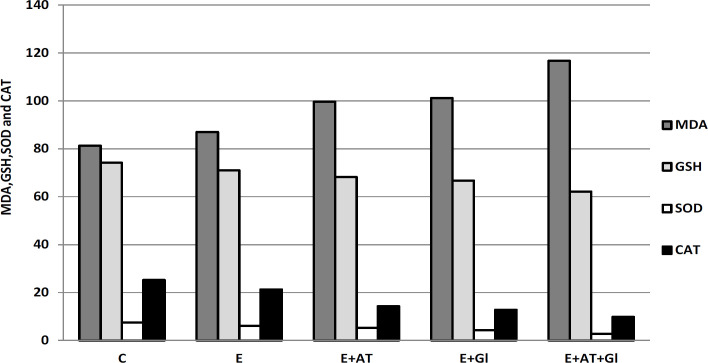
Effect of AT and/or Gl on Lipid Peroxidation and Antioxidant Enzymes in Tumor Tissue

MDA, GSH, SOD and CAT activities in solid tumor tissue are represented in (Table 2) and (Figure 2). The data revealed that solid tumor tissue of mice bearing tumor showed an increase in MDA while a decrease in GSH, SOD and CAT was recorded as compared to the normal group. Whereas, mice bearing tumor treated with AT or Gl recorded elevation in tumor Malondialdehyde (MDA) accompanied by a decline in GSH, SOD and CAT activities in comparison to the Ehrlich group. Moreover, animals bearing tumor treated with both AT and Gl revealed high elevation in tumor Malondialdehyde (MDA) accompanied by a detectable decline in GSH, SOD and CAT activities in comparison to the Ehrlich group than in each treatment alone.

*Histopathological investigation*

**Figure 3:**
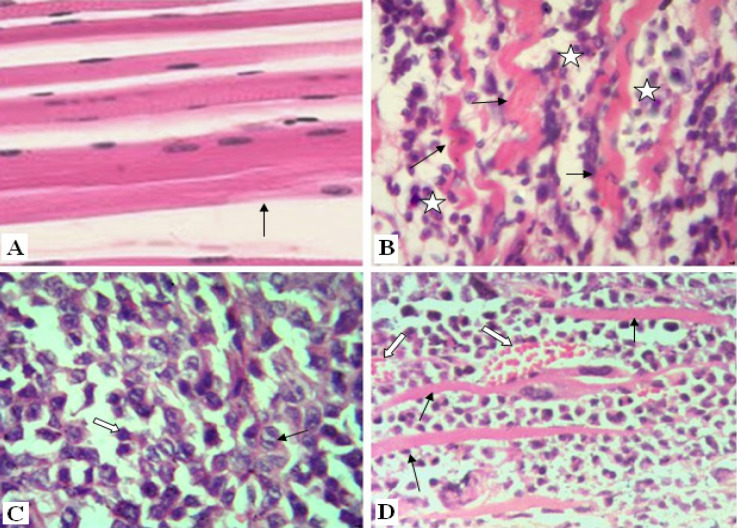
Photomicrographs of Solid Tumor Section Showing: [A]: Normal skeletal muscles (arrow). (H&E X400). [B]: Aggregation of tumor cells (star) in between necrotic ruptured muscles (arrow). (H&E X400). [C]: tumor cells are round, large with polygonal shape (white arrow), mitosis in some tumor cells (black arrow) (H&E X600). [D]: Blood capillary (white arrow) involving tumor cells in between ruptured muscles (black arrow). (H&E X400).

**Figure 4:**
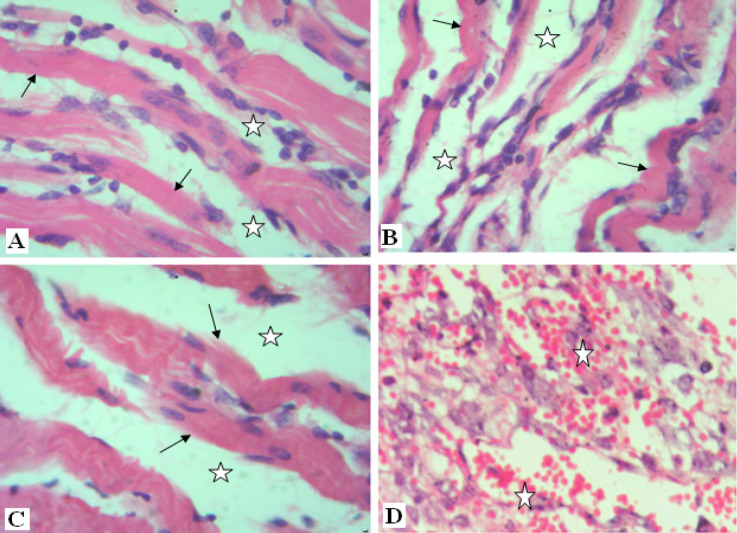
Photomicrographs of Solid Tumor Section [A]: treated with AT showing reduction in tumor cells (star) surrounding muscles (black arrow) [B]: treated with Gl showing few tumor cells (star) surrounding muscles (black arrow) [C]: treated with AT and Gl showing high attenuation in tumor cells (star) between muscles (black arrow). [D]: treated with AT and Gl showing extravasation of blood vessels between tumor cells (star) (H&E× 400).

Normal skeletal muscles are characterized by their regular parallel bundles of muscle fibers with peripheral nuclei (Figure 3A). High aggregation of tumor cells in between necrotic ruptured muscles in the Ehrlich group is markedly noticed in (Figure 3B). These tumor cells are distinguished by their round, large, polygonal shape and mitotic activity (Figure 3C). Moreover, new blood capillaries are formed “angiogenesis” between tumor cells (Figure 3D). In contrast, solid tumor treated group (E+AT) and (E+Gl) groups revealed few aggregations of tumor cells among muscular fibers (Figure 4A and 4B). Whereas, both treatment of AT and Gl against solid tumor manifested more attenuation in tumor cells (Figure 4C), with extravasation of blood vessels between tumor cells (Figure 4D).

*Immunohistohistochemical investigations*

*Angiogenic Marker (VEGF protein)*

**Figure 5:**
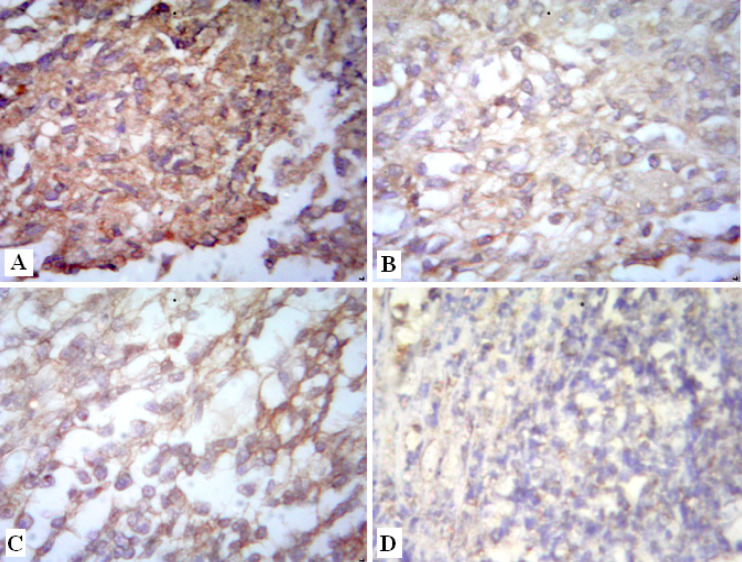
Photomicrographs of VEGF Immunostaining in Solid Tumor Section Showing: [A]: Ehrlich mice bearing tumor. [B]: Ehrlich mice bearing tumor treated with AT. [C]: Ehrlich mice bearing tumor treated with Gl. [D]: Ehrlich mice bearing tumor treated with AT and Gl

In the present study, mice bearing tumor treated with AT or Gl showed mild reduction of VEGF immunoreactivity (Figure 5B and 5C) compared to the Ehrlich group (Figure 5A). On the contrary, animals treated with both AT and Gl revealed high attenuation of VEGF immunostainig (Figure 5D) compared to the Ehrlich group (Figure 5A). Brown color indicates *VEGF* positive expression in the cytoplasm of tumor tissue. These results are also confirmed by immunohistochmical quantitative percentage in (Figure 8) and (Table 3).

*Apoptotic markers*

*Cytochrome-c protein*

**Figure 6:**
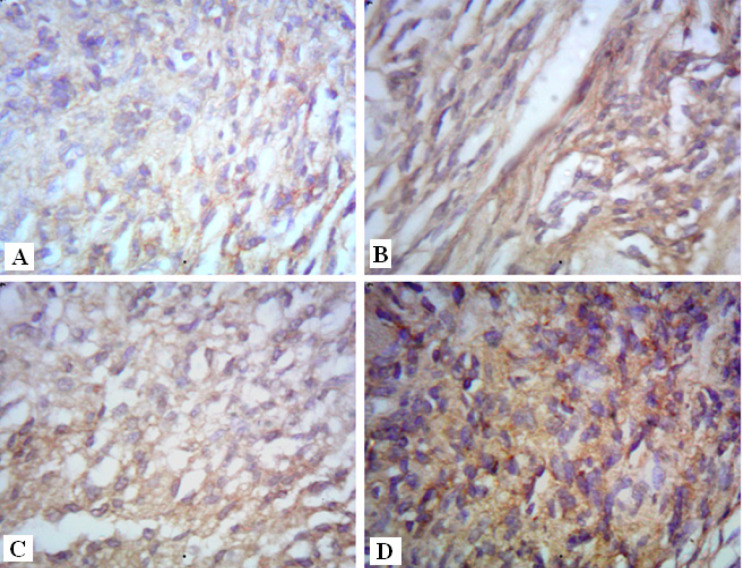
Photomicrographs of Cytochrome-c Immunostaining in Solid Tumor Section Showing: [A]: Ehrlich mice bearing tumor. [B]: Ehrlich mice bearing tumor treated with AT. [C]: Ehrlich mice bearing tumor treated with Gl. [D]: Ehrlich mice bearing tumor treated with AT and Gl

The amounts of cytochrome-c was slightly increased in mice bearing tumor treated with AT or Gl (Figure 6B and 6C) compared to the Ehrlich group (Figure 6A).On the contrary, overexpression increase of cytochrome-c was detected in mice treated with both AT and Gl (Figure 6D) compared to the Ehrlich group (Figure 6A). These results are also indicated by immunohistochmical quantitative percentage in (Figure 8) and (Table 3).

*Caspase-3 protein*

**Figure 7:**
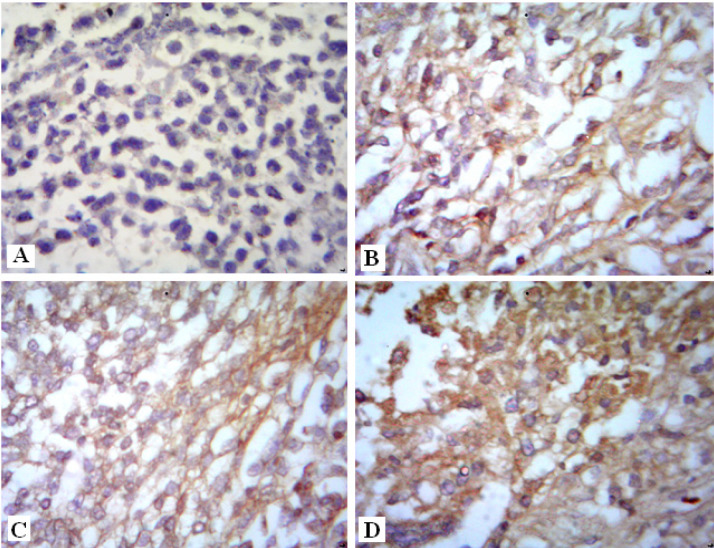
Photomicrographs of Caspase-3 Immunostaining in Solid Tumor Section Showing: [A]: Ehrlich mice bearing tumor. [B]: Ehrlich mice bearing tumor treated with AT. [C]: Ehrlich mice bearing tumor treated with Gl. [D]: Ehrlich mice bearing tumor treated with AT and Gl

**Figure 8:**
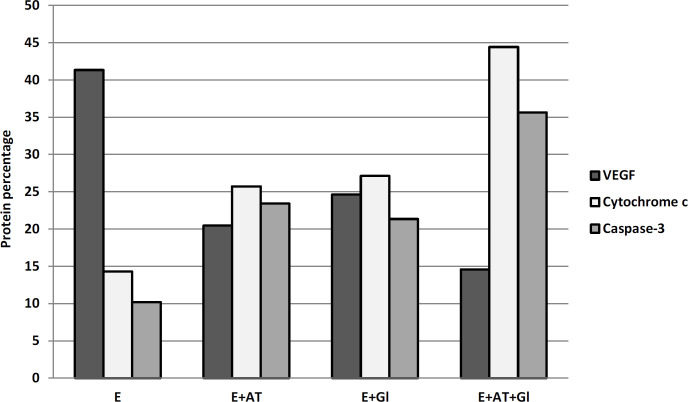
Quantitative Percentage of VEGF, Cytochrome-c and Caspase-3 Expressions in Tumor Tissue

Immunohistochemical staining of caspase-3 in mice solid tumor treated with AT or Gl revealed mild elevation (Figure 7B and 7C) compared to the Ehrlich group (Figure 7A). Whereas, the Ehrlich group treated with both AT and Gl showed Strong immunoreactivity in caspase-3 (Figure 7D) compared to the Ehrlich group (Figure 7A). These results are also demonstrated by immunohistochmical quantitative percentage in (Figure 8) and (Table 3).

**Table 1:** Effect of AT and/or Gl on Tumor Volume (mm^3^)

Tumor volume				
Groups	E	E+AT	E+Gl	E+AT+Gl
Tumor Volume (mm^3^)	252.37±1.67^a^	132.59±2.32^b^	130.96±1.51^b^	106.26±1.08^c^

Each value represents the mean ± SE (n=6). Values with different superscript letters are considered significantly different at p< 0.05. Values having the same letters are not significant to each other while values having different letters are significant to each other.

**Table 2:** Effect of AT and/or Gl on Lipid Peroxidation and Antioxidant Enzymes in Tumor Tissue

Groups	MDA (μmol/g tissue)	"GSH (mg/g tissue)"	"SOD (μg/g tissue)"	CAT (μmol/g tissue)
C	81.31±0.76^a^	74.23±0.75^a^	7.51±1.68^a^	25.21±1.52^a^
E	87.03±1.91^b^	71.02±1.11^b^	6.12±2.51^b^	21.33±2.11^b^
E+AT	99.61±1.12^c^	68.25±1.42^c^	5.26±1.93^c^	14.31±1.61^c^
E+Gl	101.11±2.66^c^	66.71±2.35^c^	4.37±1.41^c^	12.84±2.37^c^
E+AT+Gl	116.71±1.87^d^	62.14±1.53^d^	2.81±2.24^d^	9.88±1.07^d^

Each value represents the mean ± SE (n=6). Values with different superscript letters within the same raw are considered significantly different at p< 0.05. Values having the same letters are not significant to each other while values having different letters are significant to each other.

**Table 3:** Quantitative Percentage of VEGF, Cytochrome-c and Caspase-3 Expressions in Tumor Tissue

Groups	E	E+AT	E+Gl	E+AT+Gl
VEGF	41.34±0.91^a^	20.47±0.52^b^	24.65±0.31^b^	14.58±0.66^c^
cytochrome-c	14.32±0.14^a^	25.71±1.22^b^	27.12±0.87^b^	44.42±0.73^c^
caspase-3	10.21±041^a^	23.44±1.38^b^	21.34±1.57^b^	35.65±1.11^c^

Each value represents the mean ± SE (n=6). Values with different superscript letters within the same raw are considered significantly different at p< 0.05. Values having the same letters are not significant to each other while values having different letters are significant to each other

## Discussion

Several studies have attempted to estimate the proportion of common risk of cancer cases. Many factors are known to increase the risk of cancer diseases including, dietary factors, tobacco use ,certain infections, lack of physical activity, exposure to radiation, obesity, and environmental pollutants (Anand et al., 2008). At their earliest stages, tumors lack blood vessels of their own; they take their nutrients from the surrounding tissue. Cells at the tumor’s periphery get more of those nutrients than cells at the tumor core, thus most small tumors grow at their edges, while starving their cores. Cells in the tumor core liberate proteins to signal their oxygen-starved state. These proteins diffuse outward until they reach nearby blood vessels, where they stimulate the growth of new blood vessels that can supply the tumor with oxygen and other nutrients to sustain its rapid cell replication and growth. Angiogenesis is the growth of new blood vessels and is one of the features of cancer. Angiogenic blood vessels supply tumors with nutrients allowing their growth (Grossman and McNeil, 2012). Statins including Atorvastatin are commonly used to inhibit cholesterol synthesis, and to lower blood lipid levels protecting against cardiovascular diseases. These medications have also antitumor effects (Oesterle et al., 2017). Ganoderma lucidum is a kind of mushroom, which is known as Reishi in Japan. (Cao et al., 2012). Ganoderma lucidum is usually used for the prevention and treatment of several diseases, such as many forms of cancers (Joseph et al., 2011). One of its most promising activities is anti-cancer effects (Ooi and Lu, 2000). Currently, AT and Gl manifested more tumor cells attenuation against solid tumor than each treatment alone. The present study showed that the effect of Atorvastatin and/or Ganoderma lucidum resulted in a remarkable reduction in tumor size compared to the ehrlich control. These results are in accordance with Hereher et al., (2018) and Kabel et al., (2013). Free radicals accelerate the lipid peroxidation process in the cell where Malondialdehyde (MDA) is one of its final products acting as a marker of oxidative stress in cancerous cells (Gaweł et al., 2004). Glutathione (GSH), superoxide dismutase (SOD) and catalase (CAT) are endogenous antioxidant enzymes and the first line defense in the body against free radicals generated from oxidative stress (Ighodaro and Akinloye, 2019). There are many indications showing that anticancer drugs elevate ROS production in different types of cancer cells (Park et al., 2008). In the current study, mice bearing tumor treated with AT and /or Gl recorded high elevation in tumor MDA whereas high reduction of GSH, SOD and CAT activities in comparison to the Ehrlich group. These results are in accordance with Ahmed et al., (2018) who demonstrated the anticancer effect of chitosan nanoparticles in increasing MDA level and reducing the intracellular antioxidant level of GSH in tumor tissue. Yue et al., (2008) illustrated that, Ganoderma lucidum induced cytotoxicity by ROS generation and apoptosis in tumor cells, leading to increase DNA damage causing apoptosis. Also, Jones et al., (2017) showed that Atorvastatin performed to inhibit cancer proliferation by producing cellular stress. The stress induced by Atorvastatin led to an increase in cellular ROS production, which was accompanied by increased mitochondrial dysfunction leading to apoptosis in the ovarian cancer cell lines. Excess reactive oxygen species (ROS) such as superoxide anion (•O–), hydrogen peroxide (H2O2), hydroxyl ion (OH•) and malondialdehyde (MDA) are produced which cause oxidative damage to cell membranes and biomolecules. ROS also decreases the activities of CAT and SOD (Berni et al., 2019). Thus, this study indicated that free radical species were generated by AT and /or Gl which reduced intracellular antioxidant levels in tumor cells. Greenaway et al., (2016) proved that the inhibition of the mevalonate pathway using HMG-CoA reductase inhibitors could decrease levels of mevalonate and its downstream products, and thus, may have significant inhibitory influences on cancer cell growth. The level of HMG-CoA reductase has been associated with carcinogenesis and tumor progression in several cancers (Altwairgi., 2015). The histopathological study of animals bearing tumor illustrates high aggregation of tumor cells between muscular fibers which are characterized by several morphological changes; they are round, large and polygonal cells, with hyperchromatic nuclei, pleomorphic shapes and binucleation. These results are in agreement with Ahmed et al., (2018). In addition, detected angiogenesis is activated by the presence of irregular blood capillaries for supplying tumor tissue (Fodor et al., 2019). Currently, AT and/or Gl manifested an anticancer effect against solid tumor. Sections revealed attenuation in tumor cells surrounding muscular tissue; extravasations of blood capillaries causing angiogenesis. Similar results are obtained by Kubatka et al., (2011) who illustrated high regression of tumor tissue by AT in mammary tumor tissue and by Wang et al., (2020) who also showed great reduction of tumor tissue by Gl in Lung tumor tissue. The immunohistochemical study showed the elevation of apoptotic activity of cytochrome-C and caspase-3 proteins while the decrease of the angiogenic activity of VEGF protein in mice bearing tumor treated with AT and or Gl in solid tumor cells was revealed. These results agree with Bayat et al., (2018) who reported that Atorvastatin has potent anti-angiogenic and apoptotic effects through the down-regulation of VEGF, and Bcl-2, and the up-regulation of *caspase-3* expression. It has been confirmed that angiogenesis is responsible for tumor growth (Wang et al., 2009). The increase of VEGF plays an essential role in angiogenesis (Kessenbrock et al., 2010). Furthermore, most reports are in agreement that hypoxia protects cancer cells from apoptotic cell death (Brahimi-Horn et al., 2012). Intratumor hypoxia occurs when the oxygen supply is not sufficient from the blood stream which is necessary to support the growth of tumor cells. Consequently, hypoxia triggered angiogenesis which becomes abnormally active. Hypoxia increases the levels of anti-apoptotic member proteins, such as Bcl-2, whereas it decreases the levels of pro-apoptotic Bax (Palladino et al., 2012). Although mitochondrial activity is decreased in hypoxia, this organelle plays a vital role in resistance to apoptosis (Mazure et al., 2011). the decrease in oxygen induces over expression of hypoxia-inducible factor 1 (*HIF-1*) which causes inactivation of caspase-9 and caspase-3, increases anti-apoptotic Bcl-2 level and decreases pro-apoptotic Bax level (Lee et al., 2019). Also, hypoxia induces vascular endothelial growth factor (VEGF) in tumor cells and induces matrix metalloproteinase (*MMP*) expression in endothelial cells, leading to angiogenesis and tumor cell invasion (Dong, 2019).Certain therapeutic strategies are needed to be applied as a mono-treatment or in combination with chemotherapeutic drugs to overcome hypoxia and angiogenesis. Zhou et al., (2016) suggested that statins utilization may counteract hypoxia-mediated resistance to certain treatments. It was suggested that the statin inhibited invasion and metastasis in the aggressive breast cancer cell line via blockage of the mevalonate pathway (Coimbra et al., 2010) and inhibition of both MMP-2 and MMP-9. The matrix metalloproteinases (MMPs) plays significant roles in tumor growth, angiogenesis, and metastasis by the degradation of collagen and other extracellular matrix components (Klein et al., 2004). Wang et al., (2016) found that statins inhibited angiogenesis by their direct effect on tumor Adenosine monophosphate-activated protein kinase (AMPK) signaling, which inhibits downstream hypoxia-inducible factor-1α (HIF-1α)-induced angiogenesis. Thus, Atorvastatin can effectively inhibit *VEGF* expression in cancer cell. Bayat et al., (2018) proved that angiogenesis is vital in tumor growth to acquire adequate blood supply. Thus, inhibition of angiogenesis could be beneficial for tumor therapy. These results suggest that AT could be used as an anti-angiogenic agent in cancer cell. Statins induce also apoptosis by decreasing the mitochondrial transmembrane potential, increasing the activation of caspase-9 and caspase-3 inducing cell-cycle arrest (Fujiwara et al., 2017). Ganoderma lucidum treatment upregulated the level of Bax and downregulated the level of Bcl-2, resulting in an increase in the ratio of Bax/Bcl-2 in cancer cells (Sohretoglu and Huang, 2018). There are two distinct pathways that initiate apoptosis, designated as the mitochondrial and death receptor pathways (Sun et al., 2011). The mitochondrial pathway can be activated by diverse stress signals, such as toxins, reactive oxygen species, and genotoxic stress, resulting in collapse of MMP. Collapse of MMP causes the release of cytochrome-c and auto-activation of caspase-9, eventually triggering apoptosis. Death receptor-mediated pathways is the interaction of the cell surface receptors with their ligands to activate the downstream effectors (caspase-8), finally leading to apoptosis. The hallmarks of the mitochondrial-mediated intrinsic apoptotic pathways, caspases 3 and 9, were activated by Ganoderma lucidum treatment (Sohretoglu and Huang, 2018). Statins inhibit angiogenesis through their ability to downregulate vascular endothelial growth factor (VEGF), which is the major angiogenic mediator and also a tumor growth promoter (Dulak and Jozkowicz, 2005). Ganoderma lucidum extract suppresses prostate-cancer dependent angiogenesis by inhibiting the secretion of VEGF via TGF-β1supression (Sun et al., 2014). VEGF controls a variety of endothelial cell functions involved in angiogenesis and protects endothelial cells from apoptosis (Ferrara, 2004). TGF-β1transforming growth factor induced endothelial cell expression of *VEGF*, which mediates the angiogenic activity (Bostrom et al., 2004). Zhang et al., (2019) proved that Atorvastatin also induced apoptosis in both cell lines, in which the reactive oxygen species (ROS)-related mitochondrial apoptotic signaling might be involved, with increase of ROS and Bax/Bcl-2 ratio, loss of mitochondrial membrane potential (MMP), release of cytochrome-c into cytosol, and activation of Bax/caspase-9/caspase-3/PARP pathway. Atorvastatin caused G2/M cell cycle arrest with cyclinB1 and cdc2 downregulation (Zhang et al., 2019). Khan et al., (2016) demonstrated that mitochondria play a vital role in proliferation and apoptosis of a cell. In contrast to death receptor pathway, mitochondrial apoptosis pathway is initiated by various non-receptor mediated stimuli. Bcl-2 family proteins are considered the main players of mitochondrial apoptosis. Finally, it could be suggested that the combination treatment of AT and Gl is more efficient than each alone in inhibiting tumor proliferation. The tumor growth is controlled through their antiangiogenic effect by suppressing the angiogenic marker VEGF and stimulating the production of apoptotic markers cytochrome-c and caspase-3 and also by increasing oxidative stress.

## Author Contribution Statement

None.
